# MyD88 Deficiency Markedly Worsens Tissue Inflammation and Bacterial Clearance in Mice Infected with *Treponema pallidum*, the Agent of Syphilis

**DOI:** 10.1371/journal.pone.0071388

**Published:** 2013-08-05

**Authors:** Adam C. Silver, Dana W. Dunne, Caroline J. Zeiss, Linda K. Bockenstedt, Justin D. Radolf, Juan C. Salazar, Erol Fikrig

**Affiliations:** 1 Section of Infectious Diseases, Department of Internal Medicine, Yale University School of Medicine, New Haven, Connecticut, United States of America; 2 Section of Comparative Medicine, Yale School of Medicine, New Haven, Connecticut, United States of America; 3 Section of Rheumatology, Department of Internal Medicine, Yale University School of Medicine, New Haven, Connecticut, United States of America; 4 Department of Medicine, University of Connecticut Health Center, Farmington, Connecticut, United States of America; 5 Department of Pediatrics, University of Connecticut Health Center, Farmington, Connecticut, United States of America; 6 Department of Genetics and Developmental Biology, University of Connecticut Health Center, Farmington, Connecticut, United States of America; 7 Department of Immunology, University of Connecticut Health Center, Farmington, Connecticut, United States of America; 8 Division of Pediatric Infectious Diseases, Connecticut Children’s Medical Center, Hartford, Connecticut, United States of America; 9 Howard Hughes Medical Institute, Chevy Chase, Maryland, United States of America; Columbia University, United States of America

## Abstract

Research on syphilis, a sexually transmitted infection caused by the non-cultivatable spirochete *Treponema pallidum*, has been hampered by the lack of an inbred animal model. We hypothesized that Toll-like receptor (TLR)-dependent responses are essential for clearance of *T. pallidum* and, consequently, compared infection in wild-type (WT) mice and animals lacking MyD88, the adaptor molecule required for signaling by most TLRs. MyD88-deficient mice had significantly higher pathogen burdens and more extensive inflammation than control animals. Whereas tissue infiltrates in WT mice consisted of mixed mononuclear and plasma cells, infiltrates in MyD88-deficient animals were predominantly neutrophilic. Although both WT and MyD88-deficient mice produced antibodies that promoted uptake of treponemes by WT macrophages, MyD88-deficient macrophages were deficient in opsonophagocytosis of treponemes. Our results demonstrate that TLR-mediated responses are major contributors to the resistance of mice to syphilitic disease and that MyD88 signaling and FcR-mediated opsonophagocytosis are linked to the macrophage-mediated clearance of treponemes.

## Introduction

Syphilis is a multistage, sexually transmitted illness caused by the obligate human pathogen *Treponema pallidum* subsp. *pallidum* and characterized by protean clinical manifestations [1], [2], [3]. Following inoculation, usually in the genital region, spirochetes replicate locally, inducing an inflammatory response that results in the distinctive, painless chancre of primary syphilis. Within weeks, the chancre heals, indicating the local clearance of *T. pallidum*, by which time spirochetes have disseminated to various tissues and organs [1], [2], [3]. Secondary syphilis, resulting from the hematogenous dissemination of organisms, typically occurs six to eight weeks after infection. This stage of the disease most commonly involves the skin, mucous membranes and lymph nodes but can affect virtually any organ including the central nervous system [3], [4], [5]. *T. pallidum* is thought to be cleared by macrophages via antibody-mediated opsonophagocytosis [5], [6], [7], [8], [9]. Infection is contained but often not eliminated – spirochetes have the capacity to persist for years at sites of dissemination without causing symptoms [2], [3], [6]. For unclear reasons, approximately one-third of patients with latent infection develop one of the recrudescent forms of disease, collectively known as tertiary syphilis [2], [3], [6].

Despite its global importance as a human pathogen, little is known regarding the pathogenesis of syphilis and the strategies *T. pallidum* employs to evade the cellular and humoral responses it elicits within its obligate human host [2], [6], [7]. While several animal models for syphilis have been described over the years, the rabbit model has been the most widely used [8], [10], [11], [12]. In addition to being extremely susceptible to treponemal infection [13], *T. pallidum*-infected rabbits develop histopathologic changes, serologic responses, and gross lesions partially resembling those of humans [6], [8], [10], [11], [12]. Nevertheless, the use of outbred animals, which cannot easily be genetically manipulated, poses serious limitations, which are compounded further by the lack of reagents for studying inflammatory processes in rabbits. In the mid-twentieth century, investigators reported that mice could be infected with *T. pallidum* and that spirochetes persist within inoculated mice; however, unlike rabbits, symptomatic infection was not observed [10], [14], [15], [16]. In 1980, Klein and colleagues [17] reported that certain mouse strains develop cutaneous lesions after *T. pallidum* inoculation, an observation that was enhanced in the presence of ionizing radiation; however, these findings have never been reproduced [14]. The lack of clinical lesions, coupled with the low burdens of treponemes in infected tissues, has led to the widespread belief that mice are not suitable hosts for studying syphilis pathogenesis.

In other mouse models of spirochetal infection (*e.g.* murine infection with the Lyme disease spirochete *Borrelia burgdorferi*), innate immunity has been shown to be critical for limiting pathogen burdens [18], [19], [20]. Herein, we report that MyD88-deficient mice infected with *T. pallidum* have markedly worsened inflammation and a disparity in the nature of the infiltrate compared to wild-type (WT) mice. MyD88-deficient mice possess a severe defect in clearance of *T. pallidum*, and *ex vivo* studies suggest that this defect can be attributed to an inability of MyD88-deficient macrophages to phagocytose spirochetes despite the production of opsonic antibodies. Our results demonstrate that MyD88-dependent Toll-like receptor (TLR)-mediated responses contribute to the intrinsic resistance of mice to syphilitic disease. They also support the longstanding notion that opsonophagocytosis by macrophages is essential for clearance of treponemes and reveal a previously unsuspected link between MyD88 signaling and FcR-mediated phagocytosis.

## Results

### *Treponema pallidum* Elicits Cellular and Humoral Responses in Wild-type Mice Similar to those Previously Observed in Human Infection

**Figure 1:**
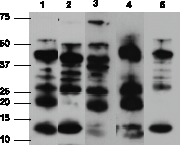
Humoral response to *T. pallidum* antigens in MyD88^−/−^ and WT mice. Sera from mice were collected 21 and 84 days post-challenge and immunoblotted against treponemal lysates for *T. pallidum*-reactive IgG abs. Representative immunoblots are shown: lane 1, day 21 MyD88^−/−^ serum; lane 2, day 21 WT serum; lane 3, day 84 MyD88^−/−^ serum; lane 4, day 84 WT serum; lane 5, serum from an immune rabbit infected with *T. pallidum* for at least 60 days.

**Figure 2:**
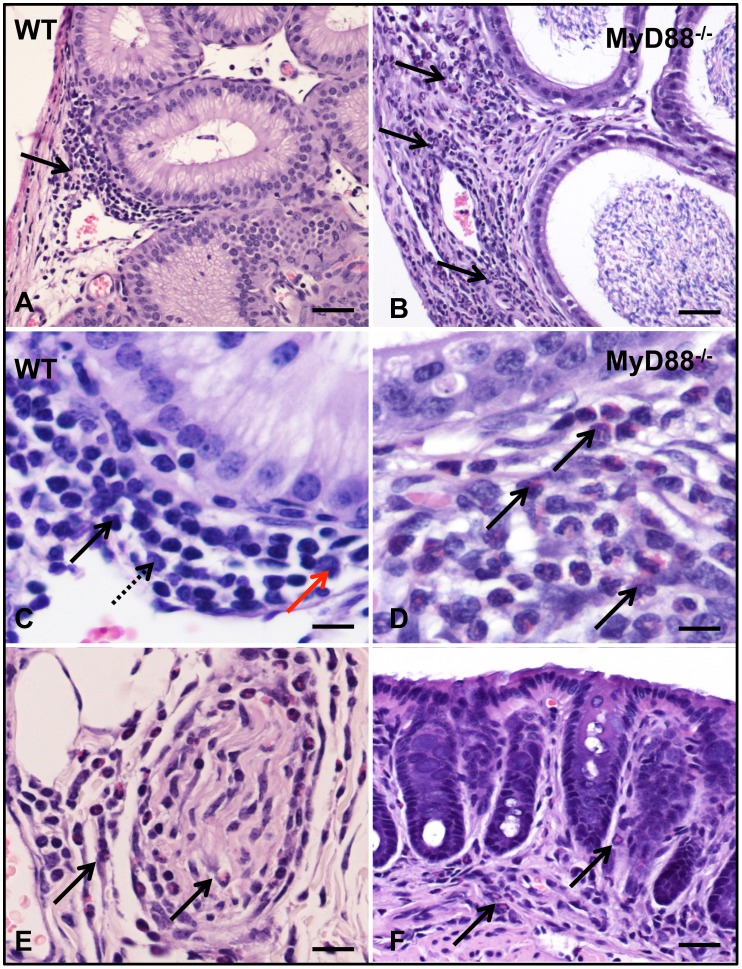
Cellular composition of the inflammatory infiltrates in MyD88^−/−^ and WT mice at 21 days post-inoculation. Both WT (A,C) and MyD88^−/−^ (B,D) mice develop interstitial inflammation of the epididymis (arrows). Inflammatory infiltrates in WT mice are localized and predominantly lymphohistiocytic (C; solid arrow = lymphocyte; dashed arrow = macrophage; red arrow = plasma cell). In contrast, MyD88^−/−^ mice display more extensive inflammatory infiltrates (arrows, B) consisting predominantly of neutrophils (solid arrows, D). Neutrophilic infiltrates were seen within the subcutis and perineurium of the skin (arrows, E) and within the submucosa of rectum (arrows, F), but not in WT mice. Sections were stained with hematoxylin and eosin; Bar = 20 µm (A,B,F), µm (C,D) and 10 µm (E).

To evaluate the utility of a murine model for experimental syphilis, we first set out to confirm and expand previous work involving WT mice [10], [14], [15], [16], [21]. Our total inoculum (1×10^8^ organisms) was slightly higher than that used previously [10], [14], [15], [16], [21] and was delivered at four sites (intragenital, intrarectal, intraperitoneal, and intradermal) in each animal in order to maximize the likelihood of establishing infection. Consistent with previous findings [10], [14], [15], [16], [21], all mice appeared healthy throughout the duration of the experiment (*i.e*. without wasting or mortality), and none developed grossly apparent skin lesions at sites of inoculation or distally. Interestingly, none of 5 mice tested, 42 days after infection, produced VDRL antibodies, which also was consistent with previous findings [22]. Immunoblot analysis has been used extensively to characterize the antibody responses of humans and experimentally infected animals against *T. pallidum* proteins [8]. As shown in Figure 1, we observed antibody profiles similar to those previously described in *T. pallidum-*infected mice, rabbits, and humans [8], [21], [23], [24], [25], [26]; responses to the well-characterized 15- and 17-kDA lipoproteins, which appear as the two low molecular weight bands, 12- and 14-kDa, respectively (Figure 1), and 47-kDA lipoprotein (TpN15, TpN17, and TpN47), were particularly prominent [27], [28]. Inflammation in WT mice was typically observed in the epididymis (Figure 2A), corpus cavernosum, rectum, and skin adjacent to the inoculation site. In both humans and rabbits, cellular infiltrates in syphilitic lesions typically consist of lymphocytes, macrophages, and plasma cells [8], [12]. We observed a similar inflammatory response in WT mice, which peaked 21 days after inoculation and subsequently subsided (Figure 2A, 2C and Table S1).

**Figure 3:**
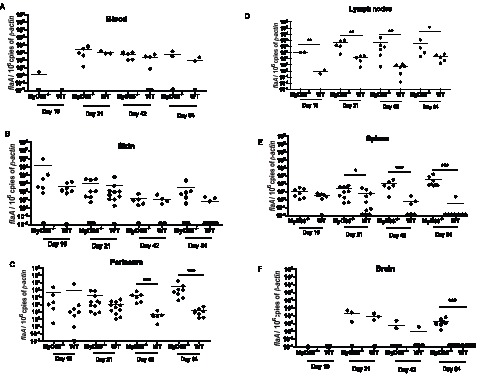
*T. pallidum* burdens in MyD88^−/−^ and WT mice. Spirochete numbers are represented as *flaA* copies per 10^6^ copies of mouse *β-actin*. Shown are bacterial burdens in blood (A), skin (B), perineum (C), lymph nodes (D), spleen (E), and brain (F) at days 10, 21, 42, and 84 post-infection. Horizontal lines represent mean values (*P<0.05, **P<0.01, ***P<0.001). Each data point represents results from one mouse; results are pooled from three independent experiments. Data points located on the horizontal-axis indicate values were below the threshold for detection (see Materials and Methods).

We next used quantitative PCR (qPCR) to assess *T. pallidum* burdens in blood, skin, spleen, perineum (genital and rectal sites), lymph nodes, and brain, 10, 21, 42, and 84 days after inoculation. We detected approximately 10^2^ to 10^3^ *flaA* copies per 10^6^ *β-actin* in blood 21 to 84 days after inoculation (Figure 3A), suggesting that the animals were spirochetemic throughout the entire time course of the infection. Pathogen burdens in skin adjacent to the intradermal injection site and perineum peaked at 10 and 21 days post-inoculation and decreased thereafter (Figure 3B and 3C). The lymph nodes contained approximately 1 *flaA* copy/10^6^ *β-actin* at days 10 and 42 post-inoculation, and 100 *flaA* copies/10^6^ *β-actin* at days 21 and 84 post-inoculation (Figure 3D). Interestingly, every perineal and lymph node sample contained detectable spirochetal DNA (Figure 3C and 3D). *T. pallidum* burdens were the lowest in the spleen and brain; in these tissues mean *T. pallidum* burdens were below 10 (spleen) and 100 (brain) *flaA* copies/10^6^ *β-actin* at all time points. Many samples contained undetectable levels of DNA, including all samples at the 84-day time point (Figure 3E and 3F).

Since our quantitation of *T. pallidum* was based on DNA detection, we next wanted to verify the viability of *T. pallidum* in mice through rabbit infectivity testing (RIT) [29], [30]. Eighty-four days after initial infection, lymph nodes were isolated from WT mice, homogenized, and injected into rabbit testes. Lymph nodes were chosen because they contained high spirochete burdens, as determined by qPCR, (Figure 3D) and are distant from the initial sites of inoculation. Four weeks after inoculation, the rabbits were sacrificed and their testes examined for the presence of viable treponemes by darkfield microscopy. Three of four rabbits had treponemes visualized by darkfield microscopy in testicular samples.

### MyD88-deficient Mice have Increased Spirochete Burdens Compared to WT Animals

MyD88^−/−^ mice were infected as above to elucidate the role of TLR-dependent responses in clearance of treponemes and generation of a local inflammatory response. As shown in Figure 1, when comparing the murine profiles to that of the infected rabbit serum, the profile of anti-treponemal antibodies in sera from MyD88^−/−^ animals did not differ appreciably from that of WT mice. Differences in spirochete burdens between the two genotypes were assessed by qPCR in blood, skin, spleen, perineum, lymph nodes, and brain at 10, 21, 42, and 84 days after inoculation. We did not detect significant differences in pathogen burdens in blood or skin adjacent to the inoculation sites of MyD88^−/−^ and WT mice at any of the time points analyzed (Figure 3A and 3B). In the perineum, MyD88^−/−^ and WT mice had similar levels of *T. pallidum* DNA at days 10 and 21 post-inoculation, while MyD88^−/−^ mice had markedly (>10^3^-fold) greater levels at the later time points (Figure 3C). Pathogen burdens were significantly higher in lymph nodes from MyD88^−/−^ mice compared to WT mice at 10, 21, 42, and 84 days post-inoculation (Figure 3D). When we compared mean *T. pallidum* DNA levels in the spleen over time, we discovered that MyD88^−/−^ mice had significantly higher bacterial loads at days 21, 42, and 84 post-inoculation (Figure 3E). As in the perineum, these differences were more pronounced at the later time points (10^2^-and 10^3^-fold greater at days 42 and 84). We did not detect *T. pallidum* DNA in the brains from any of the WT mice at day 84 but detected small amounts of *T. pallidum* DNA (average of 20 copies of *flaA*/10^6^ *β-actin*, SEM = 7.4) in the brains from all of the MyD88^−/−^ mice at this interval (Figure 3F).

We next wanted to validate the differences in spirochete burden that were observed via qPCR (Figure 3) through RIT. Compared to the normal appearing testes in rabbits inoculated with lymph nodes from WT mice, testes inoculated with lymph nodes from day 84 MyD88^−/−^ mice were grossly enlarged, had little to no sperm present, and were heavily vascularized (data not shown). All four of the rabbits injected had treponemes visualized by darkfield microscopy and they contained an approximate 100-fold greater burden when compared to WT testicular samples as determined via darkfield microscopy. We also observed significantly higher *T. pallidum flaA* RNA levels in rabbit testicles inoculated with lymph nodes from MyD88^−/−^ mice compared to rabbit testicles inoculated with lymph nodes from WT mice (Figure S1).

**Figure 4:**
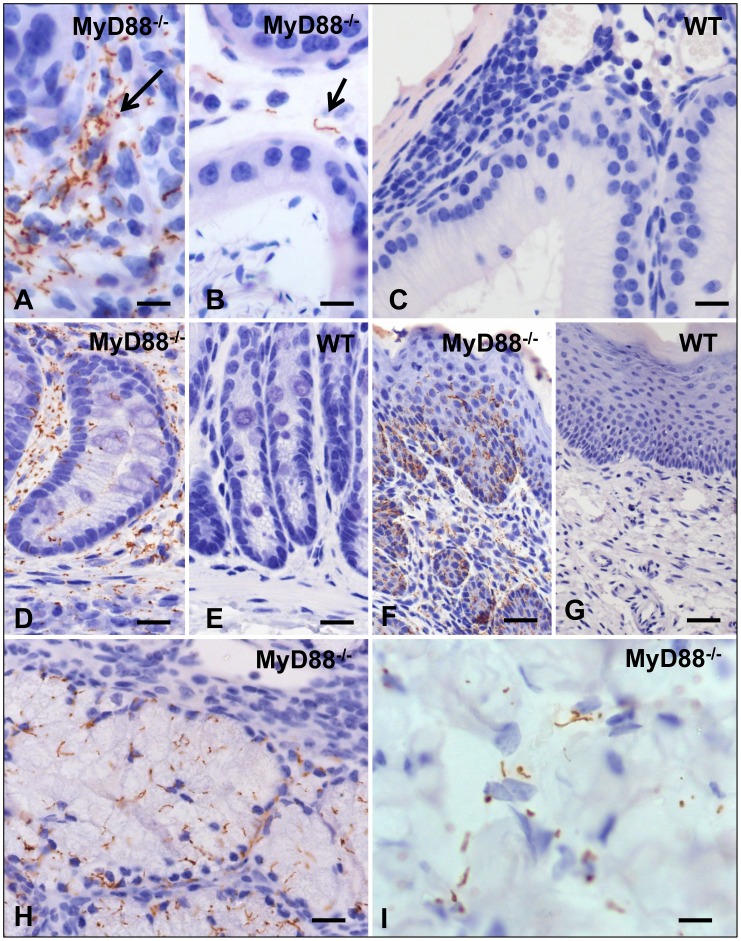
Detection of spirochetes by immunohistochemistry in tissues from MyD88^−/−^ but not WT mice. In the epididymis (A–C; 21 days post inoculation (DPI)), spirochetes are present in large numbers in inflamed foci of MyD88^−/−^ mice (arrow, A) and occasionally in non-inflamed regions (arrow, B). No spirochetes are visualized even in inflamed regions of WT mice (C). In the rectum from a MyD88^−/−^ mouse (D; 84 DPI) the interstitium is swollen with an inflammatory infiltrate containing many spirochetes; numerous spirochetes also are evident within the rectal epithelium. In contrast, the rectum of the WT animal (E; 21 DPI) is normal. Spirochetes are evident within the submucosa and in deeper layers of the vaginal epithelium of the MyD88^−/−^ (F; 84 DPI) but not in the WT (G; 84 DPI) mouse. Spirochetes are present in the epithelium of the bulbourethral gland (H; 84 DPI) and myovascular tissue of the corpus cavernosum (I; 10 DPI) in MyD88^−/−^ mice. Bars = 5 µm in panels A,B,I; 10 µm in panels C and H; and 20 µm in panels D–G.

**Figure 5:**
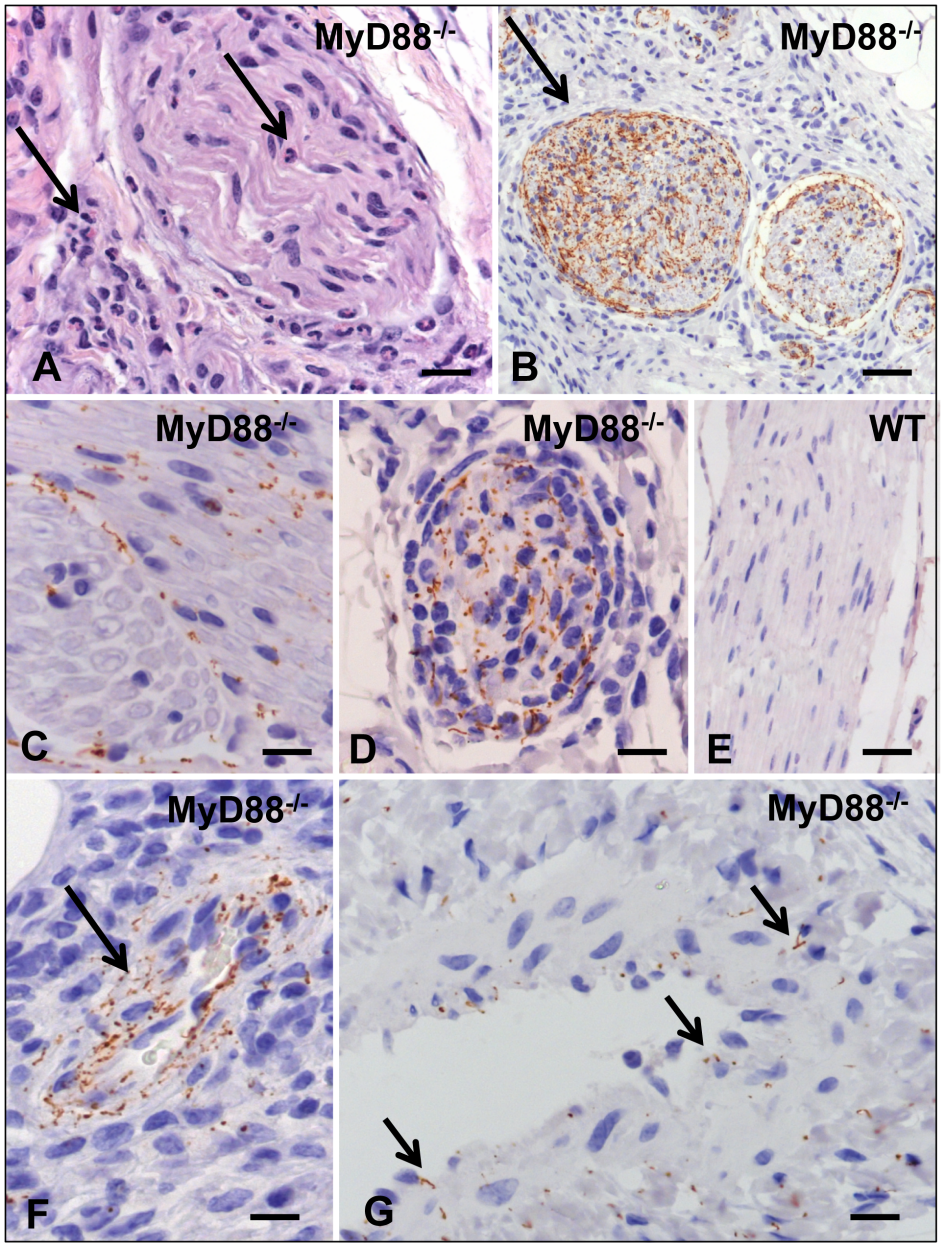
Neuronal and vascular lesions in MyD88^−/−^ mice. Hematoxylin and eosin staining showing neutrophilic inflammation in perineurium and endoneurium of a perineal nerve from a MyD88^−/−^ mouse (arrows, A; 21 DPI). Abundant spirochetes are evident by immunohistochemistry within perineal nerves (arrow, B; 21 DPI and C; 42 DPI) and rarely skin (D; 21 DPI) despite the absence of other cutaneous lesions in MyD88^−/−^ mice. Spirochetes were never seen in nerves of WT mice (E; 42 DPI). In MyD88^−/−^ mice, spirochetes are present within the wall of a rectal vessel (arrow, F; 84 DPI) and the media of a perineal arteriole (arrows, G; 21 DPI). Bars = 20 µm in panels A,B,E and 10 µm in panels C,D,F.

We next confirmed our findings of heightened pathogen burdens in MyD88^−/−^ mice by immunohistochemistry (IHC). Spirochetes were detected in approximately half (15 of 32) of MyD88^−/−^ mice compared to only 1 of 35 WT mice (Figure 4 and Table S1). Spirochetes were detected in the epididymis (Figure 4A and 4B), rectum (Figure 4D), vagina (Figure 4F), bulbourethral gland (Figure 4H) and corpus cavernosum (Figure 4I) of MyD88^−/−^ mice. Strikingly, abundant spirochetes were discovered within perineal nerves (Figure 5B and 5C) and skin (Figure 5D) of MyD88^−/−^ mice, but were never observed in nerves or skin of WT mice (Figure 5E). Spirochetes were also detected within the wall of a rectal vessel (Figure 5F) and perineal arteriole (Figure 5G) in MyD88^−/−^ mice. Vascular invasion by spirochetes was a rare finding in MyD88^−/−^ mice and was never observed in WT mice. Spirochetes were not visualized in the lymph nodes, spleen, brain, or testes in either murine background. The absence of visualized spirochetes in the lymph nodes, despite the elevated number of spirochetes detected in the via qPCR in both murine backgrounds (Figure 3D), could be due to an inclusion of sections that lack spirochetes or the possibility that residual DNA from non-intact spirochetes was detected by qPCR and contributing to the high treponemal levels.

### MyD88-deficient Mice Differ from WT in Immunopathology after Spirochete Infection

Marked overall differences in histopathology also were observed in the cellular infiltrates associated with *T. pallidum* infection in MyD88^−/−^ and WT mice. First, inflammation was more extensive in MyD88^−/−^ mice, as it was detected in 66% (21 of 32) of the knockout animals as compared to 37% (13 out of 35) of WT mice (Table S1). Secondly, infiltrates in MyD88^−/−^ mice contained a strong neutrophilic component (Figure 2B, 2C, and 2D–F) but in WT mice consisted principally of lymphocytes, macrophages, and plasma cells (Figure 2A and 2C). Lastly, the prevalence of inflammation peaked 21 days after challenge and receded thereafter in WT but MyD88^−/−^ mice demonstrated inflammation that persisted through day 84, the end of the experimental period (Table S1). Inflammation throughout the course of the study was limited to reproductive organs, rectum, and skin adjacent to the primary site of infection.

In contrast with the few secondary follicles in spleens of WT mice, those of MyD88^−/−^ mice displayed marked splenic lymphoid hyperplasia characterized by larger follicles, secondary follicle formation and marked expansion of the marginal zone (Figure S2). Extramedullary hematopoiesis was present in some animals, particularly in mice with extensive inflammation of reproductive and perineal tissues. In contrast to the WT animals, multiple large and coalescing germinal centers were evident in the spleens of MyD88^−/−^ mice 84-days post inoculation (Figure S2A and S2B). Immunostaining of T cells in periarteriolar lymphoid sheaths was comparable between genotypes (Figure S2C and S2D). In the spleens of WT mice, immunostaining identified B cells predominantly in mantle and marginal zones compared to the spleens of MyD88^−/−^ mice, in which B cells were present not only in mantle and marginal zones but also in hyperplastic germinal centers and the periarteriolar lymphoid sheath (PALS) region (Figure S2E and S2F).

### MyD88^−/−^ Bone Marrow Derived Macrophages are Defective in Opsonophagocytosis of *T. pallidum*

**Figure 6:**
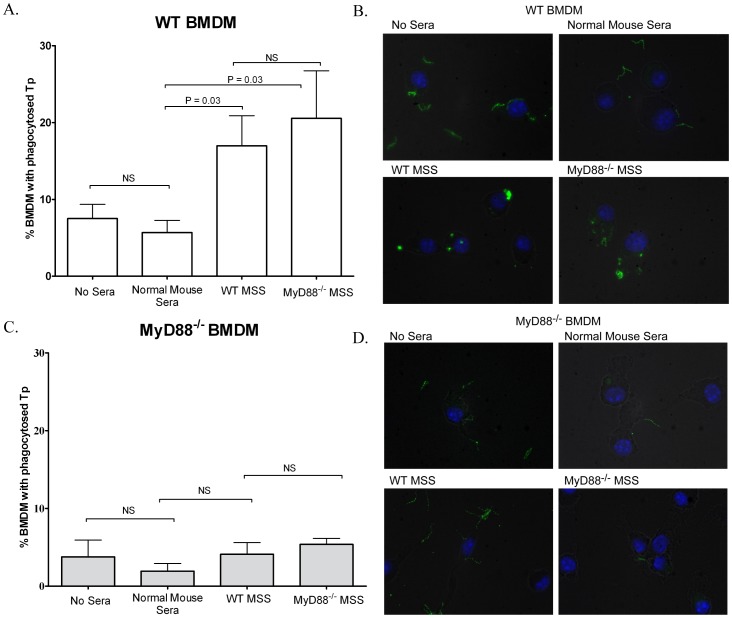
Bone marrow derived macrophages (BMDMs) contain phagocytosed *T. pallidum*. WT and MyD88 deficient BMDMs were incubated with live *T. pallidum* (MOI 30) for 6 h using four different conditions. No sera, uninfected WT mouse sera, infected WT mouse syphilitic sera and infected MyD88^−/−^ mouse syphilitic sera (infected mouse syphilitic sera was obtained 84 days post-infection). *T. pallidum u*ptake was visualized by indirect immunofluorescence microscopy as described in Materials and Methods. Bar graphs show the percentage of BMDMs that contain internalized *T. pallidum* for WT (A) and MyD88 (C) for each of the four conditions studied. Corresponding micrographs reveal representative micrographs for WT (B) and MyD88^−/−^ (D) BMDMs. Fluorescent blebs are indicative of internalized and degraded spirochetes. Uninternalized spirochetes were often visualized in close contact with the outer surface of the macrophages. *P*-values for comparisons between the different conditions studied are shown above the corresponding bar.

It is well accepted that opsonophagocytosis by macrophages is critical for treponemal clearance [5], [6], [7], [8], [9]. The finding that *T. pallidum*-infected MyD88^−/−^ mice exhibited greater bacterial loads than their WT counterparts prompted us to ascertain whether MyD88^−/−^ mice failed to produce opsonic antibodies and/or were defective in their capacity to internalize opsonized *T. pallidum*. As shown in Figure 6A and 6B, sera from both WT and MyD88^−/−^ mice obtained 84 days post-inoculation markedly enhanced uptake of *T. pallidum* by WT bone marrow-derived macrophages (BMDMs). These results demonstrate that both mouse genotypes produce opsonic antibodies in response to infection with *T. pallidum*. Surprisingly, in contrast to the WT BMDMs, neither WT nor MyD88^−/−^ syphilitic sera enhanced uptake of treponemes by MyD88^−/−^ BMDMs (Figure 6C and 6D). In parallel experiments, we demonstrated that spirochetal uptake by the WT BMDMs, when incubated with sera from either WT or MyD88^−/−^ infected mice, was associated with increased production of both TNF-α and IL-10 when compared to those incubated with normal mouse sera (Figure S3). As expected, MyD88^−/−^ cells incubated with *T. pallidum* under any condition (i.e., unstimulated, normal mouse sera, sera from infected WT or MyD88^−/−^ mice) failed to produce either cytokine (data not shown).

## Discussion

*T. pallidum* are cleared from the primary site of inoculation in the mammalian host, but can persist in diverse tissues [1], [2], [3]. Spirochete burdens are modulated, in part, by macrophages via antibody-mediated opsonophagocytosis [5], [6], [7], [8], [9]. Despite the dramatic reduction of organisms, *T. pallidum* can endure for years without causing symptoms [2], [6]. The mechanisms that allow a few treponemes to escape host responses remain elusive. To help discern the immune mechanisms that control spirochete numbers and/or contribute to immunopathogenesis, an animal model is required in which immunologic reagents are readily available and genetic manipulation of the immune system is routine. While the disease manifestations and immunological response to infection in rabbits somewhat mimics human syphilis [10], [11], [12], this model is not ideal for detailed studies to dissect the immunobiology of infection. Therefore, we first confirmed and expanded older studies describing *T. pallidum* infection in mice [10], [15], [16], [22]. Our findings demonstrated that spirochetes are present in both blood and local sites of inoculation and disseminate to other tissues. Mice produced antibodies that promote phagocytosis of spirochetes by macrophages. Spirochetes also elicited inflammatory infiltrates, primarily consisting of macrophages, lymphocytes, and plasma cells at the inoculation site, partially resembling the histopathologic changes observed in human syphilitic lesions [12], [31]. These observations suggest that the immune processes elicited during spirochetal infection in humans also are induced in mice. As innate immunity is critical for the initial control of many pathogens, including some spirochetes [18], [19], [20], we examined *T. pallidum* infection in mice lacking the capacity to elicit TLR-dependent responses that signal through the MyD88-dependent pathway, with the goal of altering the infection evident in wild-type mice.

*T. pallidum* has developed novel strategies to evade host defenses. The outer membrane of *T. pallidum* is devoid of lipopolysaccharide and has a paucity of antigenic targets, compared with other spirochetes, making immediate immune recognition difficult [32], [33]. In particular, *T. pallidum* has numerous lipoproteins, which can stimulate TLR1/2 heterodimers [34], [35], [36], however, the vast majority of these lipoproteins are located beneath the outer membrane and not surface-exposed in intact, viable organisms, thereby limiting their initial detection by the host [37], [38], [39]. In addition to impeding the development of an initial innate immune response, this unique outer membrane structure also prevents efficient opsonophagocytosis, even in the presence of opsonic antibodies that eventually develop as part of acquired immunity [32], [33], [37], [38], [39], [40]. Spirochetes, therefore, initially replicate with little interference from the host; however, presumably, as *T. pallidum* multiply, some spirochetes naturally degrade and are digested, thereby triggering a response that ultimately includes the release of proinflammatory cytokines and the recruitment of additional immune cells and antibodies to the site of infection [34], [35], [36].

Over time the opsonophagocytic uptake of spirochetes and the eventual TLR-mediated activation of macrophages causes a reduction of pathogen burden and influences the nature of the inflammatory infiltrate (*i.e.* macrophages, lymphocytes, plasma cells). The similarities between pathogen burden in WT and MyD88^−/−^ mice at early time points suggest that macrophage uptake of *T. pallidum* prior to development of opsonizing antibodies is inefficient. In contrast to WT mice, the defect in phagocytic uptake of *T. pallidum* by MyD88^−/−^ mice persisted despite the development of opsonizing antibodies, as illustrated by the continued elevation in pathogen burden. While MyD88-deficient mice produced opsonic antibodies, their macrophages were severely hindered in their ability to phagocytose *T. pallidum*, which prevented their activation and the subsequent cytokine response. This altered pathogen burden also resulted in the predominance of neutrophils in the infiltrate. TLRs are vital in the phagocytosis of various organisms [18], [41], [42] and the absence of these innate immune pathogen recognition receptors or MyD88 leads to pathogen specific defects in phagocytosis that result in a reduction in microbe uptake or phagolysomal killing [43]. *T. pallidum* clearance by macrophage-mediated opsonophagocytosis, however, points to a previously unknown role for MyD88 in this Fc-receptor mediated process.

Overall, the alterations in MyD88-dependent host responses result in decreased spirochete clearance. Counter intuitively, the decrease in the functionality of these immune responses also causes an increase in inflammation, as well as an alteration in the nature of the histopathologic characterization of the lesions, which are primarily neutrophilic. The mechanisms that cause this are likely multifactorial. For example, the continued increase in *T. pallidum* load may result in continued exposure of the host to elevated levels of spirochetes, which may cause compensatory and alternative MyD88-independent pathways to initiate immune responses that contribute to inflammation without clearing the organism. The continued presence of neutrophils suggest that an early innate response is unable to evolve into a more mature lymphocytic response due to the absence of all the signals required for immune development. These studies set the stage for investigations on the relative factors that contribute to *T. pallidum* control and the immunopathogenesis of disease. Indeed, studies in TLR2-deficient mice suggest a phenotype intermediate between MyD88-deficient and control mice, indicating that TLR2 participates in the control of infection (Figure S4).

Attempts to develop a murine model of *T. pallidum* infection have been met with very limited success [14], [15], [16]. Our study differs from these earlier reports in that we used a multitude of techniques to examine, in greater detail, *T. pallidum* dissemination and persistence in mice as well as the cellular and humoral responses to the pathogen. These data demonstrate the critical role of MyD88-dependent immune responses in the control of murine *T. pallidum* infection and macrophage-driven opsonophagocytosis of the bacterium. This model should enable researchers to more clearly delineate the immunobiologic basis for disease, further characterize treponeme-host interactions, and lead to the development of new vaccines and therapeutics for syphilis.

## Materials and Methods

### Ethics Statement

This study was carried out in strict accordance with the recommendations in the Guide for the Care and Use of Laboratory Animals of the National Institutes of Health. The protocol was approved by the Institutional Animal Care and Use Committee at Yale University (#2012-10404 ) or University of Connecticut Health Center (UCHC) (#100071-0314).

### Animals

For mouse infection experiments, 8–12 week old C57BL/6 MyD88^−/−^ mice and C57BL/6J WT (Jackson Laboratories) were maintained on antibiotic-free chow in animal facilities at the Yale School of Medicine. For isolation of BMDMs (conducted at UCHC), C57BL/6 MyD88^−/−^ mice were kindly provided by Dr. Egil Lien at the University of Massachusetts with permission from Dr. S. Akira, Osaka, Japan. Sex and age-matched WT C57BL/6 mice were purchased from Harlan Laboratories. Rabbit studies were conducted using seronegative 6–8 pound New Zealand white rabbits (Charles River Laboratories) house and cared for at the Yale University School of Medicine and the UCHC.

### Propagation of *Treponema pallidum*

The Nichols-Farmington strain of *T. pallidum* [44] was propagated in rabbits as previously described [29]. Briefly, 5×10^8^ *T. pallidum* were injected into each testicle of the rabbit. The rabbits were housed in a BL2 room kept at 62–65°F and maintained on antibiotic-free rabbit chow. 11–12 days post-inoculation, animals were euthanized and testes aseptically removed. Spirochetes were extracted in RPMI on a rotary shaker. The concentration of *T. pallidum* was determined by placing a 10-µl aliquot of a 1/500 dilution into a Petroff-Hausser chamber for enumeration by darkfield microscopy. The concentration of the treponemal suspension was adjusted to 3–4×10^8^ organisms/ml in RPMI. Stocks of organisms were prepared by freezing aliquots in RPMI with 20% NRS and 10% glycerol.

### Inoculation of Mice

Mice were inoculated intradermally (between the scapulae), intraperitoneally, intrarectally and intragenitally (females, intravaginally; males, percutaneously in the corpus cavernosa) with 2.5×10^7^ organisms per site (1×10^8^ total organisms). Intra-rectal and vaginal inoculations were performed with a gavage-type needle. Mice were evaluated every other day for signs of cutaneous or systemic infection. Groups of mice were sacrificed at day 10, 21, 42, and 84 days post-inoculation. Skin adjacent to the inoculation site, genital (testicles/penis/epididymis in males, ovary/fallopian tubes/uterus in females), rectal, lymph node (inguinal/brachial/axillary), splenic, and brain tissues were harvested for analyses as described below.

### Rabbit Infectivity Testing

Rabbit infectivity testing was performed as previously described [44], [45]. Briefly, mouse inguinal, brachial, and axillary lymph nodes were collected and placed in a sterile Petri dish containing 1–2 ml of saline and 20% NRS. The tissue was finely minced and compressed with forceps into the liquid. Spirochetes were extracted further by placing dish on a platform rotator for 10 min. 1 ml of the fluid extract was drawn up in a 3-cc Luer-lok syringe. Rabbits were anesthetized with 3–5 mg/kg (20 mg/ml) xylazine followed by 35–50 mg/kg of ketamine (100 mg/ml). Once sedated, testes were cleansed with alcohol and one testis was inoculated with the 1 ml fluid extract while the contralateral testis served as a control. Four weeks after injection, the rabbit was euthanized, testes were harvested, and spirochetes were extracted as described above for examination by darkfield microscopy and qRT-PCR.

### Isolation of DNA

Tissue samples were homogenized using the Bullet Blender® (Next Advance) and DNA was extracted using a DNeasy blood and tissue kit (Qiagen) according to the manufacturer’s instructions.

### Quantitative PCR

Primers and TaqMan probes used for *T. pallidum flaA* and mouse *β-actin* were previously described [44]. qPCR reactions were performed in 10 µl reaction volumes containing 2.5 µl of extracted DNA, 1X TaqMan Gene Expression Master Mix (Applied Biosystems), 0.2 µM of each sense and antisense primers, and 0.2 µM probe. Molecular grade deionized water was used as a no-template control. qPCR was performed using a real-time PCR 7500 Fast System (Applied Biosystems). A threshold cycle (Ct) value greater than 38 for the *flaA* amplification was interpreted as an absence of treponemal DNA and a value of zero was entered into the analysis. When feasible, on days of animal sacrifice, an uninfected WT mouse was sacrificed, its tissues collected, and DNA extracted as a negative control to assess the possibility of cross-contamination; all such negative control reactions had undetectable levels of *flaA*. Analyses were performed using a standard curve. The standard curve for *flaA* analysis was performed as previously described [45]. For normalization, a fragment of the mouse *β-actin* gene was amplified using previously described primers [46] and cloned into the pCR2.1 cloning vector (Invitrogen Inc.). Values are expressed as copies of *flaA* per 10^6^ copies of mouse *β-actin*.

### Immunoblot Analysis

Freshly harvested treponemes were centrifuged at 10,000×*g* for 20 min at 4°C and washed once with cold PBS. The pellet was resuspended in *T. pallidum* lysis buffer (1x Laemmli sample buffer supplemented with 8 M urea and 5% β-mercaptoethanol) to yield approximately 1×10^8^ treponemes per µl. An aliquot of the resuspended lysate was incubated at 95°C for 10 min and approximately 1×10^8^ organisms were loaded in alternating lanes of a 4–15% Mini-PROTEAN^®^ TGX™ polyacrylamide gel (molecular markers were loaded in the remaining wells) followed by standard SDS-PAGE. The proteins were transferred to a polyvinyl difluoride (PVDF) membrane using a mini-trans blot system (BioRad Laboratories) at 25 volts for 20 min. Following transfer, the membrane was cut into 10 mm×75 mm strips, which were placed in individual troughs in blotting trays and incubated overnight at 4°C with mouse serum diluted 1∶500 in 5% skim milk. The strips were washed three times with 0.1% Tween-20 in PBS (10 min each wash with rocking) and then incubated with an anti-mouse HRP antibody at 1∶20,000 for 45 min in 0.1% Tween-20 in PBS. The strips were then washed five times as described above and chemiluminescence detection was performed using the ECL immunoblot detection kit (Amersham).

### Histopathology and Immunohistochemistry

Tissues were fixed in formalin and stained with hematoxylin and eosin or subjected to immunohistochemical staining with anti-*T. pallidum* Abs (Biocare, Concord, CA).

### Opsonophagocytosis of Treponemes by Murine Macrophages

BMDMs were recovered by flushing femurs and tibias of WT or MyD88^−/−^mice with DMEM and then incubating retrieved cells in tissue culture-treated 25 cm^2^-flasks (BD Falcon, BD Biosciences, San Jose, CA) overnight at 37°C with 5% CO_2_ to eliminate adherent fibroblasts and granulocytes. Cell suspensions (1×10^7^) were transferred into a 10-cm^2^ bacteriological Petri dishes (BD-Falcon) and incubated with DMEM supplemented with 10% FBS, 20% L292-cell conditioned media, 0.01% HEPES, 0.01% sodium pyruvate, and 0.01% L-glutamine. Seven days after isolation and incubation cell monolayers were exposed to ice-cold PBS and gently removed by gentle scraping. Single cell macrophage suspensions were seeded into 6-well tissue culture-treated plates at a concentration of 1×10^6^ cells/(2 ml per well) and allowed to adhere overnight. The following day, old medium was replaced and live *T. pallidum* (MOI 30∶1) were added for a 6 h co-incubation at 37°C in 5% CO_2_ under four different stimulation conditions; (a) no mouse sera, (b) 10% heat inactivated uninfected WT mouse sera, (c) 10% heat inactivated WT mouse syphilitic sera and (d) 10% heat inactivated MyD88^−/−^ mouse syphilitic sera. All infected mouse sera used for these experiments was obtained 84 days post-spirochetal inoculation. Supernatants were removed for later assay for cytokines using a mouse Cytokine Bead Array kit (BD Biosciences, San Diego, CA). *T. pallidum*-cell (BMDMs) associations were visualized using a previously described immunofluorescence assay (IFA) (1). Rat anti-FlaA was used as the primary antibody and Alexa 488 goat anti-rat (from Invitrogen) as the secondary antibody. Cells were visualized using epifluoresence microscopy with an Olympus BX-41 microscope using a 100X (1.4NA) oil immersion objective equipped with a Retiga Exi CCD camera (Q Imaging, Tucson, AZ) and the following Omega filter sets: DAPI, and FITC. Image processing and analysis were performed using ImageJ (NIH, v1.41b) and LSM Image Browser (Zeiss, v4.2.0.121). The percentage of murine macrophages containing internalized spirochetes was quantified using a minimum of 100 WT or MyD88**^−/−^** cells for each of the four conditions studied.

### Statistical Analysis

Exponential data were transformed into Gaussian distributed linear data in Excel and differences in *T. pallidum* burdens between MyD88^−/−^ and WT mice in a particular tissue and day post-inoculation were assessed via the Students t-test using GraphPad Prism, version 4.0b. Percentages of bacterial uptake by WT and MyD88^−/−^ macrophages were compared amongst the different stimulus by using either a paired or unpaired Student *t* test or the equivalent non-parametric methods (*i.e.* Wilcoxon), using GraphPad Prism 4.0. For each experiment, both the standard deviation and the standard error of the mean were calculated. *p* values of <0.05 were considered significant.

## Supporting Information

Figure S1 **Detection of viable** ***T. pallidum*** **in rabbit testes inoculated with lymph nodes from MyD88^−/−^ and WT mice (84 DPI).** *T. pallidum* values are represented as *flaA* transcripts per 10^6^ transcripts of rabbit *β-actin*. Horizontal lines represent mean values (*p<0.05). Each data point represents results from one rabbit; results are pooled from two independent experiments. Data points located on the X-axis indicate *T. pallidum* RNA was undetectable.
(TIF)

Figure S2 **Splenic changes in MyD88^−/−^ and WT mice.** Spleens from a MyD88^−/−^ (A) and WT (B) mouse at 21 days post-inoculation. There is marked lymphoid hyperplasia with formation of germinal centers and expansion of the marginal zone in the MyD88^−/−^ mouse compared to the WT animal. In the WT mouse (D,F), the periarteriolar lymphoid sheath (D, T cell region) is surrounded by a modest mantle and marginal zone (F, B cell area). In the MyD88^−/−^ mouse, multiple germinal centers enclose the periarteriolar lymphoid sheath (C). Germinal centers and mantle/marginal zones are markedly expanded by B cells (E). Hematoxylin and eosin (A, B); CD3 (T cell marker) and B220 (B cell marker) immunohistochemistry (C–F). Bars = 100 µm in panels A and B and 50 µm in panels C–F.
(TIF)

Figure S3 **Opsonophagocytosis of live treponemes enhances cytokine production by BMDMs.** WT BMDMs were incubated for 6 h with live spirochetes (MOI:30) using three different conditions; normal mouse sera, infected WT mouse syphilitic sera and infected MyD88^−/−^ mouse syphilitic sera (infected mouse sera was obtained 84 days post-infection). (A) TNF-α and (B) IL-10 concentrations (pg/ml) were measured in the supernatants. Bars depict the means +/− standard error of the mean from four independent experiments. *P*-values for comparisons between the conditions studied are shown above the corresponding bar.
(TIF)

Figure S4 ***T. pallidum*** **burdens in MyD88^−/−^, TLR2^−/−^, and WT mice inoculated in the genitals with 2.5×10^7^ organisms.** Treponeme numbers are represented as *flaA* copies per 10^6^ copies of mouse *β-actin*. Shown are bacterial burdens in genitals (A), spleen (B), lymph nodes (C), and brain (D) at days 21, 42, 84, and 120 post infection. Horizontal lines represent mean values (*p<0.05, **p<0.01, ***p<0.001). Each data point represents results from one mouse. Results are pooled from two independent experiments. Data points located on the X-axis indicate undetectable levels of *T. pallidum* DNA.
(TIF)

Table S1 **Inflammation and spirochete load in MyD88^−/−^ and WT mice determined by immunohistochemistry.**
(DOCX)
