# Supplementary figures and images for: MyD88 Deficiency Markedly Worsens Tissue Inflammation and Bacterial Clearance in Mice Infected with Treponema pallidum, the Agent of Syphilis
Source: PLoS One. 2013 Aug 5;8(8):e71388. doi: 10.1371/journal.pone.0071388 (PMC3734110; doi:10.1371/journal.pone.0071388)

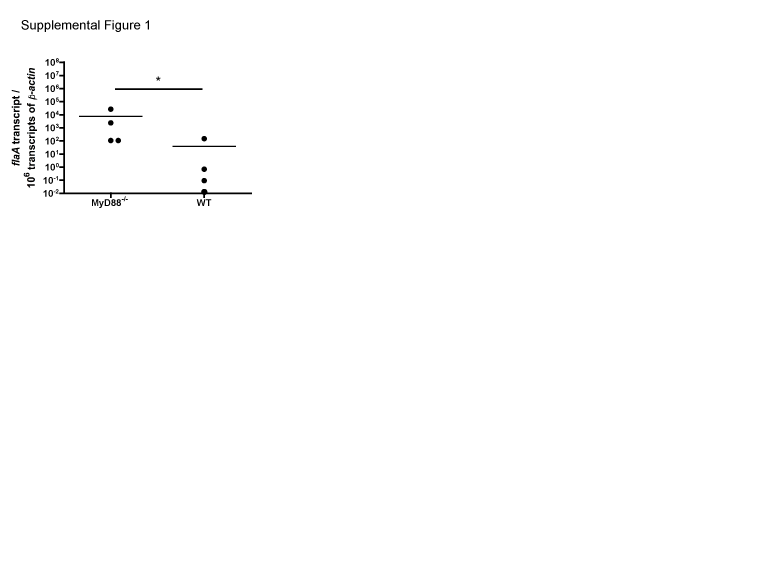

Supplement: Figure S1 — Detection of viable T. pallidum in rabbit testes inoculated with lymph nodes from MyD88−/− and WT mice (84 DPI). T. pallidum values are represented as flaA transcripts per 106 transcripts of rabbit β-actin. Horizontal lines represent mean values (*p<0.05). Each data point represents results from one rabbit; results are pooled from two independent experiments. Data points located on the X-axis indicate T. pallidum RNA was undetectable. (TIF) [file pone.0071388.s001.tif]

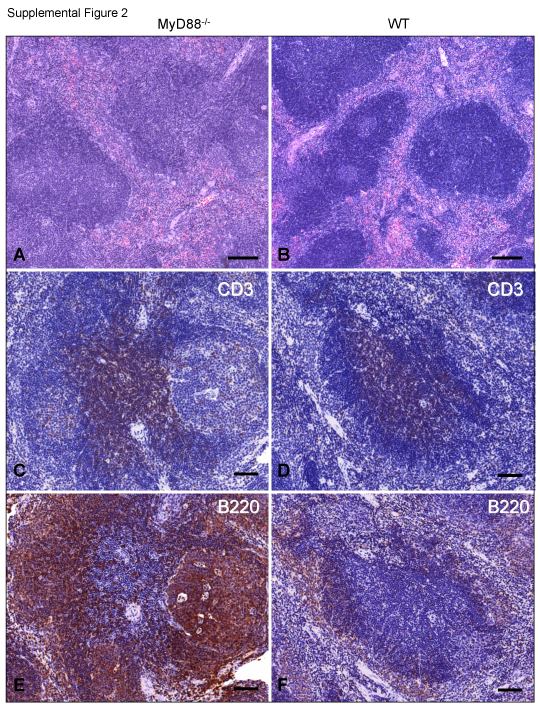

Supplement: Figure S2 — Splenic changes in MyD88−/− and WT mice. Spleens from a MyD88−/− (A) and WT (B) mouse at 21 days post-inoculation. There is marked lymphoid hyperplasia with formation of germinal centers and expansion of the marginal zone in the MyD88−/− mouse compared to the WT animal. In the WT mouse (D,F), the periarteriolar lymphoid sheath (D, T cell region) is surrounded by a modest mantle and marginal zone (F, B cell area). In the MyD88−/− mouse, multiple germinal centers enclose the periarteriolar lymphoid sheath (C). Germinal centers and mantle/marginal zones are markedly expanded by B cells (E). Hematoxylin and eosin (A, B); CD3 (T cell marker) and B220 (B cell marker) immunohistochemistry (C–F). Bars = 100 µm in panels A and B and 50 µm in panels C–F. (TIF) [file pone.0071388.s002.tif]

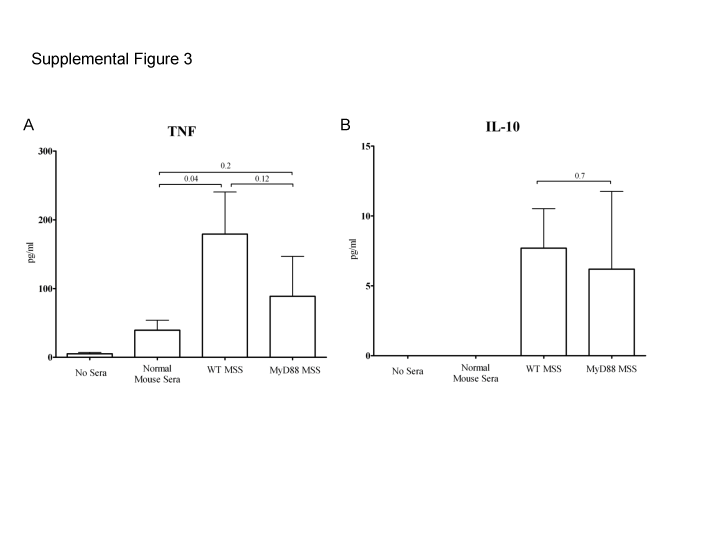

Supplement: Figure S3 — Opsonophagocytosis of live treponemes enhances cytokine production by BMDMs. WT BMDMs were incubated for 6 h with live spirochetes (MOI:30) using three different conditions; normal mouse sera, infected WT mouse syphilitic sera and infected MyD88−/− mouse syphilitic sera (infected mouse sera was obtained 84 days post-infection). (A) TNF-α and (B) IL-10 concentrations (pg/ml) were measured in the supernatants. Bars depict the means +/− standard error of the mean from four independent experiments. P-values for comparisons between the conditions studied are shown above the corresponding bar. (TIF) [file pone.0071388.s003.tif]

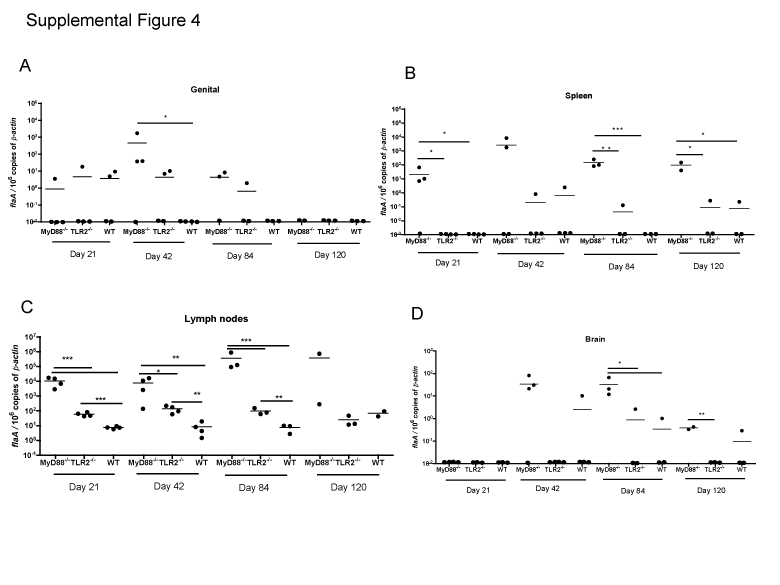

Supplement: Figure S4 — T. pallidum burdens in MyD88−/−, TLR2−/−, and WT mice inoculated in the genitals with 2.5×10^7^ organisms. Treponeme numbers are represented as flaA copies per 106 copies of mouse β-actin. Shown are bacterial burdens in genitals (A), spleen (B), lymph nodes (C), and brain (D) at days 21, 42, 84, and 120 post infection. Horizontal lines represent mean values (*p<0.05, **p<0.01, ***p<0.001). Each data point represents results from one mouse. Results are pooled from two independent experiments. Data points located on the X-axis indicate undetectable levels of T. pallidum DNA. (TIF) [file pone.0071388.s004.tif]
