# Supplementary material for: MyD88 Deficiency Markedly Worsens Tissue Inflammation and Bacterial Clearance in Mice Infected with Treponema pallidum, the Agent of Syphilis
Source: PLoS One. 2013 Aug 5;8(8):e71388. doi: 10.1371/journal.pone.0071388 (PMC3734110; doi:10.1371/journal.pone.0071388)
Supplement: Table S1 — Inflammation and spirochete load in MyD88−/− and WT mice determined by immunohistochemistry. (DOCX) [file pone.0071388.s005.docx]

| Table S1. Inflammation and spirochete load in MyD88^-/-^ and WT mice determined by immunohistochemistry. | | | | |
| --- | --- | --- | --- | --- |
|  |  |  |  |  |
|  | MyD88^-/-^ | | WT | |
| Days post infection | Inflammation*^a^* | Spirochetes*^b^* | Inflammation*^a^* | Spirochetes*^b^* |
|  |  |  |  |  |
| 10 | 4/7 | 1/7 | 2/8 | 0/8 |
| 21 | 6/11 | 5/11 | 8/14 | 1/14 |
| 42 | 4/6 | 4/6 | 2/6 | 0/6 |
| 84 | 7/7 | 5/7 | 1/7 | 0/7 |
|  | 21/32 | 15/32 | 13/35 | 1/35 |
| *^a^*Presence of inflammation detected in any tissue / total number of mice analyzed  *^b^*Presence of spirochetes detected in any tissue / total number of mice analyzed | | | | |
|  |  |  |  |  |
